# Uses and perceptions of medications among French older adults: results from the 2020 French Health Barometer survey

**DOI:** 10.1186/s12877-022-03289-9

**Published:** 2022-07-20

**Authors:** Anh Thi-Quynh Tran, Noémie Soullier, Joël Ankri, Marie Herr, Laure Carcaillon-Bentata

**Affiliations:** 1 Anti-Infective Evasion and Pharmacoepidemiology, Université Paris-Saclay, UVSQ, INSERM, CESP, 78180 Montigny-le-Bretonneux, France; 2 Santé Publique France, French national public health agency, 12 rue du Val d’Osne, 94415 Saint-Maurice, France; 3 Epidemiology and Public Health Department, AP-HP, Université Paris-Saclay, Raymond-Poincaré Hospital, 92380 Garches, France

**Keywords:** Polypharmacy, Self-medication, Perceptions, Older adults, Health survey

## Abstract

**Background:**

There are few studies reporting on self-medication, perceptions or difficulties older adults have with their medications. This study aimed to describe the uses and the perceptions of medications among older adults in France and to identify patient groups based on that information.

**Methods:**

We used data from the 2020 ‘French Health Barometer’ – a nationally-representative cross-sectional survey. We assessed polypharmacy (five or more medications), self-medication, and patient perceptions of medications. Robust Poisson regression was used to investigate socio-demographic and health-related factors associated with the outcomes. Latent class analysis was used to identify patient groups classified by the use and the perceptions of medications. Factors associated with group assignment were investigated by multinomial logistic regression. All analyses were weighted.

**Results:**

The study sample comprised 1,623 respondents aged 70–85 years. Polypharmacy and self-medication were reported in 23.5 and 48.7% of the older population, respectively. Polypharmacy was associated with increasing age, low education, and impaired health status. Self-medication was associated with female sex and high education. Among individuals taking at least 1 medication, 8.2% reported not to understand all their medications, and 9.7% having difficulty taking medications as prescribed. Among individuals taking at least 2 medications, 23.2% thought that they took too many medications. Three patient groups were identified: ‘Non*-*polypharmacy, positive perceptions’ (62.5%), ‘Polypharmacy, positive perceptions’ (28.0%), and ‘Negative perceptions’ (9.5%).

**Conclusions:**

Polypharmacy and self-medication are common in French older adults. One segment of people reported negative perceptions of their medications regardless of their polypharmacy status. This underlines the difference between the objective and perceived measures of polypharmacy.

**Supplementary Information:**

The online version contains supplementary material available at 10.1186/s12877-022-03289-9.

## Introduction

The share of older adults continues to grow in France and globally [1]. The increase in the prevalence of multimorbidity in the ageing population is further accompanied by an increase in consumption of medications [2].

The concurrent use of five or more medications is commonly referred to as polypharmacy [3]. Polypharmacy is prevalent in older adults. A study using data from 17 European countries and Israel showed that the prevalence of polypharmacy among adults aged $\ge$65 years ranged between 26.3 and 39.9% [4]. Older people with polypharmacy are at a higher risk of adverse drug events and drug-drug interactions [3]. Polypharmacy is also associated with potentially inappropriate medication use, longer hospitalization, and mortality [3, 5].

A better understanding of patient perceptions of their medications may help reduce the burden of polypharmacy. The propensity to accept deprescribing notably depends on patient beliefs and attitudes towards medications, which can be complex. The study of Rozsnyai et al. found that although 97% of Swiss older patients with polypharmacy were satisfied with their medications, 16% felt that their medications were a burden to them. [6] A good knowledge of medications may influence patient perceptions, thus contributing to better health-related behaviors such as medication adherence [7]. However, when asking older adults about the purpose of their medications in another study, it appeared that about one third had difficulty indicating the purpose of one of their medications or more and that it was partly related to polypharmacy [8].

Besides prescription medications, older adults can use nonprescription medications for chronic or temporary ailments without consulting a doctor [9]. Despite its potential benefits, self-medication with nonprescription medications involves risks such as misdiagnosis and drug-drug interactions, thereby causing detrimental health effects to older population [10]. In France, a study found that over 50% of adults living in Paris metropolitan area had self-medicated in the past four weeks [11]. However, limited data are available on the older population.

In this study, we aimed to describe medication uses (polypharmacy, self-medication) and perceptions of medications in the older population in France and to investigate their associated factors. We also sought to use information about medication usage and patient perceptions to find distinct groups among older adults taking $\ge$ 2 medications.

## Methods

### Study design and population

We used data from the ‘French Health Barometer’ survey conducted between January and March 2020 [12]. ‘French Health Barometer’ is a population-based telephone survey on health-related perceptions, attitudes, and behaviors. Random selection and weighting ensured the sample to be representative of the French-speaking population aged 18–85 years living in the community in France. Design weights, reflecting the individual selection probability (in the household and according to the individual phone equipment), were adjusted by calibration to match the target population structure in terms of age, gender, region of residency, town size, number of persons per household and education level, using data from the 2018 Labor Survey conducted by National Institute of Statistics and Economic Studies. Questions about uses and perceptions of medications were administered to respondents aged $\ge$ 70 years.

### Measurement

#### Polypharmacy

Respondents reported the number of different medications currently taken in a day. A medication could contain one or multiple active components. Polypharmacy was defined as the use of five medications or more.

#### Self-medication

Respondents were asked whether they have ever self-medicated (Regularly/Occasionally/Never). Those who answered ‘Regularly’ or ‘Occasionally’ were both coded as ‘Yes’ while those who answered ‘Never’ were coded as ‘No’. Nonprescription drugs were grouped in six categories: drugs for fever, pain, and headache/drugs for cough and sore throat/drugs for stress and insomnia/drugs for heartburn and indigestion/vitamins and minerals/others drugs.

#### Patient perceptions of prescribed medications

Respondents taking $\ge$ 1 medication were asked two questions:

- ‘Do you understand the reasons why the medications were prescribed?’ (Yes, for all the medications/Yes, but not for all the medications/No). Respondents were coded as ‘Yes’ when they answered ‘Yes, for all the medications’, otherwise coded as ‘No’.
- ‘Do you find it easy/difficult to take your medications as prescribed?’ (Easy/Sometimes difficult/Difficult). Those who answered ‘Sometimes difficult’ or ‘Difficult’ were both coded as ‘Difficult’ and asked to choose one or more of the following reasons for their difficulty: Have lots of medications to take/Run out of medications/Medications are hard to take/Changes in medication appearance/Have side effects/Other reasons.

Respondents taking $\ge$ 2 medications were asked the third question:

- ‘Do you think you take too many medications?’ (Yes/No)

#### Other variables

Socio-demographic variables included sex, age, living situation, socio-professional category, education level, and income. Socio-professional categories were defined using the French classification for occupations as follows: farmers/craftsmen and traders/intermediate professions/executive and intellectual professions/employees/manual workers [13]; retired persons were classified in their last job category. Income was regarded as the household income divided by the number of consumption units, using the Organization for Economic Co-operation and Development scale, to insure comparison of the standards of living of different size and compositions of households [14].

Health-related variables included frailty and three health variables of The Minimum European Health Module, namely self-perceived health, chronic conditions, and activity limitation [15]. Frailty was assessed according to Fried’s phenotype definition, adapted for declarative data with good performance [16, 17]. Nine questions were used to define the presence of five frailty components: involuntary weight loss, exhaustion, low physical activity, muscular weakness, and impaired mobility (Supplementary File–Table S1). Respondents having missing data on $\le$ 2 frailty components were assigned a frailty status. Frailty was determined as follows: not frail (0 components), pre-frail (1–2 components) and frail (3–5 components).

### Statistical analysis

Descriptive statistics were performed to summarize the characteristics of study population. We had five dichotomous outcomes (Yes/No): polypharmacy, self-medication, and three outcomes pertaining to patient perceptions (‘*Think they take too many medications’, ‘Do not understand all medications’, ‘Have difficulty taking medications as prescribed’*). We investigated their associated factors using robust Poisson regression. In cross-sectional studies, this method can be used to directly estimate prevalence ratios instead of odds ratios to avoid the overestimation of odds ratio when outcomes are not rare [18]. For each outcome, we built two multivariate models: Model 1 including only socio-demographic variables and Model 2 including socio-demographic and health-related variables. The number of medications taken was also added in Model 2 for the three outcomes pertaining to perceptions. Because health-related variables and the number of medications taken are potentially on the causal pathway between socio-demographic variables and our outcomes, adjustment for these variables could induce distorted associations of the outcomes with socio-demographic variables [19]. Model 1 was hence used to interpret associations of the outcomes with socio-demographic variables while Model 2 was used to interpret the associations with health-related variables and the number of medications taken. Model 1 and Model 2 of each outcome were conducted on the same sample in which there is no missing data on explanatory variables.

We hypothesized that there were unobserved groups in the study population and that individuals in these groups differed in terms of the uses and the perceptions of medications. We then used latent class analysis (LCA) to group patients based on the five outcome variables. Respondents taking $\ge$2 medications were included in this analysis. The number of groups was determined by interpretability and the Akaike information criterion. The differences among patient groups were assessed through corrected design-based F-statistic [20]. Multivariate multinomial regression models were conducted to investigate factors associated with group assignment in the same strategy to that described in the preceding paragraph.

All analyses were weighted. Analyses were conducted using STATA 14. We used poLCA package in R to perform LCA.

### Sensitivity analysis

As the missing data rate for income was around 10%, we performed sensitivity analyses using multiple imputation with chained equations to check the robustness of the results obtained from the complete case analyses. We imputed missing values for explanatory variables based on socio-demographic variables, health-related variables, polypharmacy, and self-medication. Multivariate regression models were then conducted with imputed dataset.

## Results

Among 1,623 respondents included in this study, 9 respondents did not report the number of medications taken and 3 did not report the frequency of self-medication. Among 1,321 respondents taking $\ge$ 1 medication, 4 respondents did not report their understanding of medications, and 3 did not answer the question about difficulty taking medications as prescribed. Among 1,080 respondents taking $\ge$ 2 medications, 6 respondents did not answer whether they think they take too many medications.

**Fig. 1:**
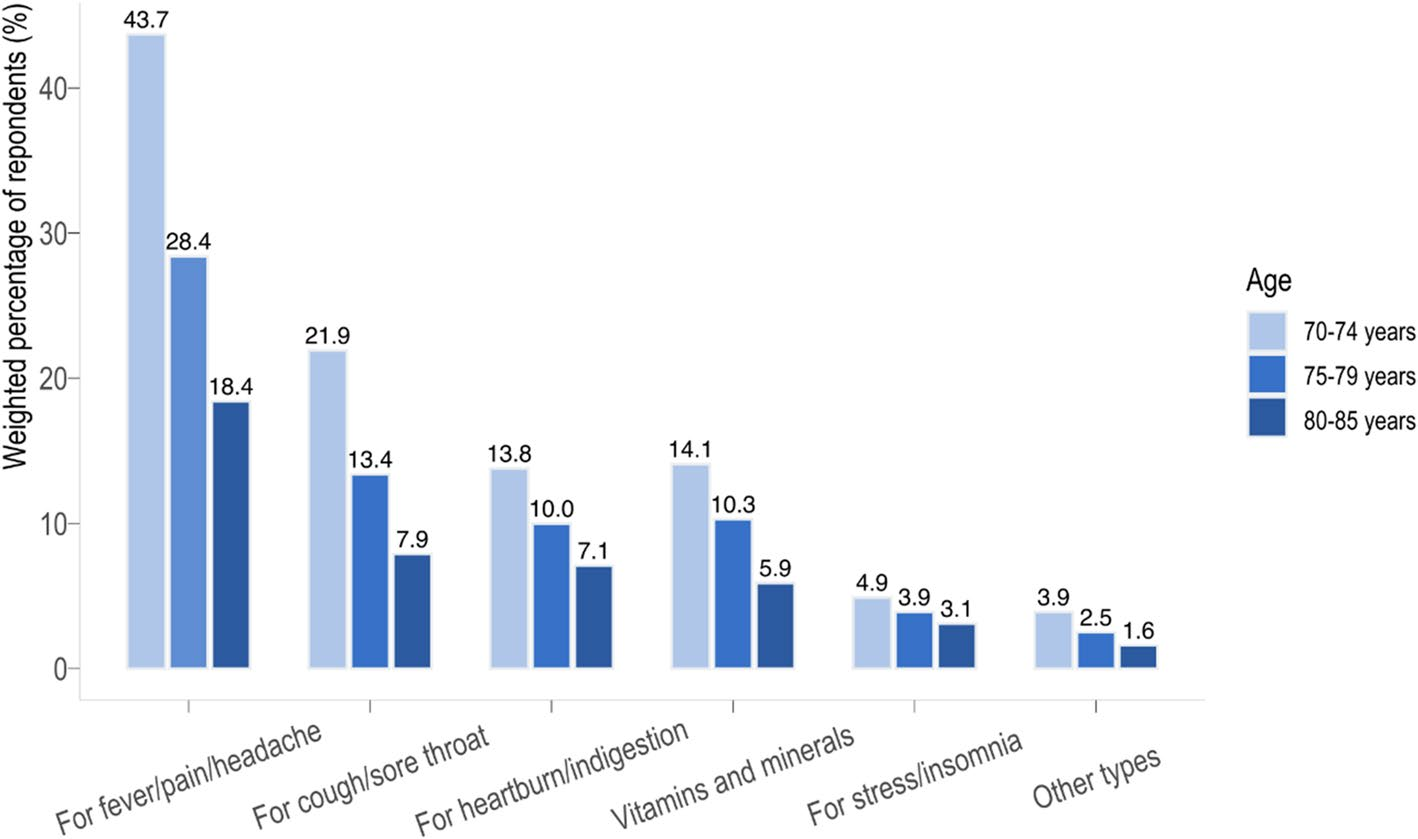
Nonprescription medication use by age groups (*N* = 862)

Table 1 shows descriptive statistics of the study sample. Among the French population aged 70–85 years, 23.5% (95%CI: 20.9–26.3%) were on polypharmacy while 16.8% (95%CI: 14.7–19.1%) reported not to take any medication. In addition, 48.7% of respondents (95%CI: 45.7–51.7%) reported self-medication (2.8% regularly and 45.9% occasionally). Among nonprescription drug categories, drugs for fever, pain, and headache were the most reported (90.7% [95%CI: 88.3–92.7%]), followed by drugs for cough and sore throat (43.1% [95%CI: 39.2–47.2%]), drugs for heartburn and indigestion (30.9% [95%CI: 27.2–34.7%]), vitamins and minerals (30.4% [95%CI: 26.9–34.2%]), drugs for stress and insomnia (11.9% [95%CI: 9.6–14.6%]), and other drugs (7.7% [95%CI: 5.9–10.0%]) (Fig. 1).

**Table 1:** Descriptive statistics (unweighted sample size, weighted percentages)

				**Think they**	**Do not**	**Have difficulty**
			**Self-**	**take too many**	**understand all**	**taking medications**
**Characteristics**	**All**	**Polypharmacy**^**(a)**^	**medication**^**(b)**^	**medications**^**(c)**^	**medications**^**(d)**^	**as prescribed**^**(e)**^
	**(*n* = 1,623)**	**(*n* = 334)**	**(*n* = 862)**	**(*n* = 248)**	**(*n* = 90)**	**(*n* = 105)**
**Sociodemographic variables**						
*Sex*						
Male	681 (44.7)	158 (24.8)	339 (45.0)	132 (27.3)	46 (7.4)	42 (7.4)
Female	942 (55.3)	176 (22.5)	523 (51.7)	116 (19.6)	44 (8.8)	63 (11.6)
*Age group (years)*						
70 – 74	789 (43.6)	123 (18.1)	447 (52.5)	126 (26.8)	34 (5.1)	43 (7.5)
75 – 79	474 (30.8)	106 (26.5)	251 (50.2)	67 (20.3)	24 (8.3)	26 (7.4)
80 – 85	360 (25.6)	105 (29.1)	164 (40.5)	55 (21.3)	32 (12.8)	36 (15.6)
*Living situation*						
Not living alone	906 (65.8)	161 (21.3)	503 (49.3)	135 (23.6)	44 (7.2)	58 (10.1)
Living alone	717 (34.2)	173 (27.8)	359 (47.5)	113 (22.5)	46 (9.8)	47 (8.9)
*Socio-professional category*						
Intermediate/Executive/Intellectual	771 (38.7)	142 (19.7)	443 (53.4)	114 (23.1)	32 (5.1)	35 (5.3)
Employees	399 (28.5)	86 (24.4)	211 (50.3)	51 (20.0)	18 (7.1))	30 (13.0)
Laborers/Farmers/Craftsmen/Traders	421 (32.8)	97 (26.1)	189 (41.7)	78 (25.0)	38 (11.9)	35 (9.4)
Missing	32					
*Education level*						
None/Primary	437 (38.9)	129 (32.2)	180 (38.4)	76 (25.1)	36 (11.7)	45 (14.7)
Secondary	685 (42.8)	133 (19.3)	366 (52.1)	110 (22.3)	35 (6.0)	34 (6.2)
Post-secondary	487 (18.3)	67 (14.2)	310 (62.7)	59 (20.9)	19 (5.5)	25 (6.7)
Missing data	14					
*Income (euros)*						
< 1500	394 (30.6)	109 (31.7)	182 (43.0)	69 (24.6)	35 (11.9)	31 (11.6)
1500 – 3000	705 (46.2)	143 (23.1)	373 (47.1)	108 (23.5)	34 (7.2)	58 (12.1)
> 3000	373 (23.2)	58 (16.8)	229 (60.3)	52 (21.5)	13 (3.7)	11 (2.7)
Missing	151					
**Health-related variables**						
*Self-perceived health*						
Very good/Good	942 (55.9)	96 (12.0)	528 (50.3)	101 (19.1)	31 (5.4)	33 (5.0)
Very bad/Bad/Fair	678 (44.1)	238 (38.2)	332 (46.5)	145 (26.6)	59 (11.0)	72 (14.6)
Missing	3					
*Chronic conditions*						
No	685 (43.3)	55 (9.6)	376 (47.7)	49 (16.0)	19 (5.9)	17 (4.7)
Yes	930 (56.7)	278 (34.1)	438 (50.0)	198 (26.5)	70 (9.3)	87 (12.3)
Missing	8					
*Activity limitation*						
Not limited	965 (59.3)	107 (12.4)	517 (48.4)	95 (17.6)	31 (4.6)	29 (4.1)
Limited but not severely	419 (25.9)	106 (31.1)	253 (58.4)	92 (29.9)	37 (13.4)	45 (15.5)
Severely limited	232 (14.8)	119 (55.4)	91 (33.7)	61 (28.2)	22 (11.6)	31 (19.0)
Missing	7					
*Frailty status*						
Not frail	710 (42.4)	78 (12.9)	396 (50.9)	72 (17.8)	21 (4.8)	19 (4.0)
Pre-frail	722 (43.5)	155 (22.3)	398 (52.2)	126 (23.7)	39 (7.9)	53 (9.9)
Frail	188 (14.1)	101 (60.0)	67 (31.4)	50 (32.7)	29 (16.8)	33 (22.7)
Missing	3					

^(a)^: Sample size without missing outcome data / Sample size with missing outcome data: 1,614 / 1,623

^(b)^: Sample size without missing outcome data / Sample size with missing outcome data: 1,620 / 1,623

^(c)^: Sample size without missing outcome data / Sample size with missing outcome data: 1,074 / 1,080

^(d)^: Sample size without missing outcome data / Sample size with missing outcome data: 1,317 / 1,321

^(e)^: Sample size without missing outcome data / Sample size with missing outcome data: 1,319 / 1,321

Of those taking $\ge$ 1 medication, 8.2% (95%CI: 6.3–10.4%) reported not to understand all their medications and 9.7% (95%CI: 7.7–12.1%) had difficulty taking medications as prescribed. ‘Have side effects’ (33.9% [95%CI: 23.5–46.1%]) was the most common barrier preventing patients from taking medications as prescribed, followed by ‘Changes in medication appearance’ (20.5% [95%CI: 12.0%–32.7%]), ‘Have lots of medications to take’ (16.1% [95%CI: 9.5–26.1%]), ‘Medications are hard to take’ (4.7% [95%CI: 1.7–12.6%]), and ‘Run out of medications’ (4.4% [95%CI: 1.8–10.2%]). Other reasons were mentioned by 24.7% (95%CI: 16.2–32.7%) of the respondents. Besides, 23.2% (95%CI: 20.2–26.5%) of the respondents taking $\ge$ 2 medications thought that they took too many medications.

Table 2 displays the factors associated with polypharmacy and self-medication. Overall, polypharmacy was positively associated with increasing age, low education, very bad/bad/fair self-perceived health, presence of chronic conditions, activity limitation, and frailty. Self-medication was positively associated with female sex, high education, and negatively associated with frail status.

**Table 2:** Factors associated with polypharmacy and self-medication

	**Polypharmacy**			**Self-medication**		
**Characteristics**		**Model 1**	**Model 2**		**Model 1**	**Model 2**
		**(*n* = 1,424)**	**(*n* = 1,424)**		**(*n* = 1,428)**	**(*n* = 1,428)**
	**Crude PR**	**Adjusted PR**	**Adjusted PR**	**Crude PR**	**Adjusted PR**	**Adjusted PR**
	**(95% CI)**	**(95% CI)**	**(95% CI)**	**(95% CI)**	**(95% CI)**	**(95% CI)**
**Sociodemographic variables**						
* Sex*						
Male	Ref	Ref	Ref	Ref	Ref	Ref
Female	0.91	0.79	0.67 ***	1.15 **	1.24 ***	1.24 ***
	(0.72 – 1.14)	(0.59 – 1.05)	(0.51 – 0.88)	(1.01 – 1.30)	(1.08 – 1.43)	(1.08 – 1.43)
* Age group (years)*						
70 – 74	Ref	Ref	Ref	Ref	Ref	Ref
75 – 79	1.46 ***	1.23	1.04	0.96	0.97	0.99
	(1.11 – 1.92)	(0.92 – 1.65)	(0.77 – 1.39)	(0.84 – 1.10)	(0.84 – 1.12)	(0.86 – 1.14)
80 – 85	1.61 ***	1.39 **	1.17	0.77 ***	0.85 *	0.87
	(1.22 – 2.13)	(1.02 – 1.87)	(0.90 – 1.52)	(0.65 – 0.92)	(0.70 – 1.02)	(0.73 – 1.05)
*Living situation*						
Not living alone	Ref	Ref	Ref	Ref	Ref	Ref
Living alone	1.31 **	1.13	0.94	0.96	1.02	1.07
	(1.05 – 1.64)	(0.86 – 1.48)	(0.72 – 1.21)	(0.85 – 1.09)	(0.87 – 1.18)	(0.92 – 1.23)
*Socio-professional category*						
Intermediate/Executive/Intellectual	Ref	Ref	Ref	Ref	Ref	Ref
Employees	1.24	0.90	0.87	0.94	1.12	1.12
	(0.93 – 1.65)	(0.64 – 1.28)	(0.62 – 1.21)	(0.82 – 1.09)	(0.94 – 1.33)	(0.95 – 1.32)
Laborers/Farmers/Craftsmen/Traders	1.32 **	0.93	0.93	0.78 ***	1.00	0.98
	(1.01 – 1.74)	(0.68 – 1.28)	(0.70 – 1.22)	(0.67 – 0.91)	(0.83 – 1.20)	(0.82 – 1.18)
*Education level*						
None/Primary	Ref	Ref	Ref	Ref	Ref	Ref
Secondary	0.60 ***	0.69 ***	0.74 **	1.36 ***	1.35 ***	1.32 ***
	(0.47 – 0.77)	(0.53 – 0.91)	(0.57 – 0.95)	(1.15 – 1.60)	(1.13 – 1.62)	(1.11 – 1.58)
Post-secondary	0.44 ***	0.51 ***	0.55 ***	1.63 ***	1.60 ***	1.53 ***
	(0.32 – 0.60)	(0.35 – 0.75)	(0.38 – 0.79)	(1.39 – 1.92)	(1.29 – 1.97)	(1.25 – 1.88)
*Income (euros)*						
< 1500	Ref	Ref	Ref	Ref	Ref	Ref
1500 – 3000	0.73 **	0.88	0.89	1.09	1.03	1.01
	(0.56 – 0.94)	(0.65 – 1.17)	(0.67 – 1.17)	(0.92 – 1.30)	(0.86 – 1.24)	(0.85 – 1.21)
> 3000	0.53 ***	0.75	0.78	1.40 ***	1.22 *	1.22 *
	(0.37 – 0.75)	(0.49 – 1.14)	(0.51 – 1.20)	(1.18 – 1.66)	(0.97 – 1.55)	(0.97 – 1.54)
**Health-related variables**						
*Self-perceived health*						
Very good/Good	Ref	-	Ref	Ref	-	Ref
Very bad/Bad/Fair	3.18 ***		1.53 ***	0.92		1.00
	(2.43 – 4.17)		(1.13 – 2.08)	(0.81 – 1.05)		(0.87 – 1.15)
*Chronic conditions*						
No	Ref	-	Ref	Ref	-	Ref
Yes	3.57 ***		2.24 ***	0.95		0.92
	(2.57 – 4.95)		(1.62 – 3.12)	(0.84 – 1.08)		(0.80 – 1.05)
*Activity limitation*						
Not limited	Ref	-	Ref	Ref	-	Ref
Limited but not severely	2.51 ***		1.70 ***	1.21 ***		1.30 ***
	(1.88 – 3.35)		(1.22 – 2.37)	(1.06 – 1.37)		(1.13 – 1.50)
Severely limited	4.46 ***		2.06 ***	0.70 ***		0.91
	(3.42 – 5.82)		(1.47 – 2.89)	(0.56 – 0.87)		(0.71 – 1.17)
*Frailty status*						
Not frail	Ref	-	Ref	Ref	-	Ref
Pre-frail	1.73 ***		1.03	1.03		1.02
	(1.26 – 2.37)		(0.74 – 1.42)	(0.91 – 1.16)		(0.90 – 1.17)
Frail	4.65 ***		1.74 ***	0.62 ***		0.68 **
	(3.45 – 6.28)		(1.21 – 2.51)	(0.48 – 0.80)		(0.50 – 0.92)

*PR*  Prevalence Ratio

^*^ p < 0.1; ** p < 0.05; *** p < *0.01*

Model 1: adjusted for socio-demographic variables

Model 2: adjusted for socio-demographic and health-related variables

Regarding the perceptions, the proportion of 70–85 years old who think that they take too many medications and the proportion of those who do not understand all their medications increased with the number of medications taken (Supplementary File–Tables S2 and S3). Females thought more often that they take too many medications than males. People aged 80–85 years and those with low income were less likely to understand their medications. Finally, having difficulty taking medications as prescribed was positively associated with increasing age (80–85 years), low income (< 1,500 euros), activity limitation, and frailty (Supplementary File–Table S4).

**Fig. 2:**
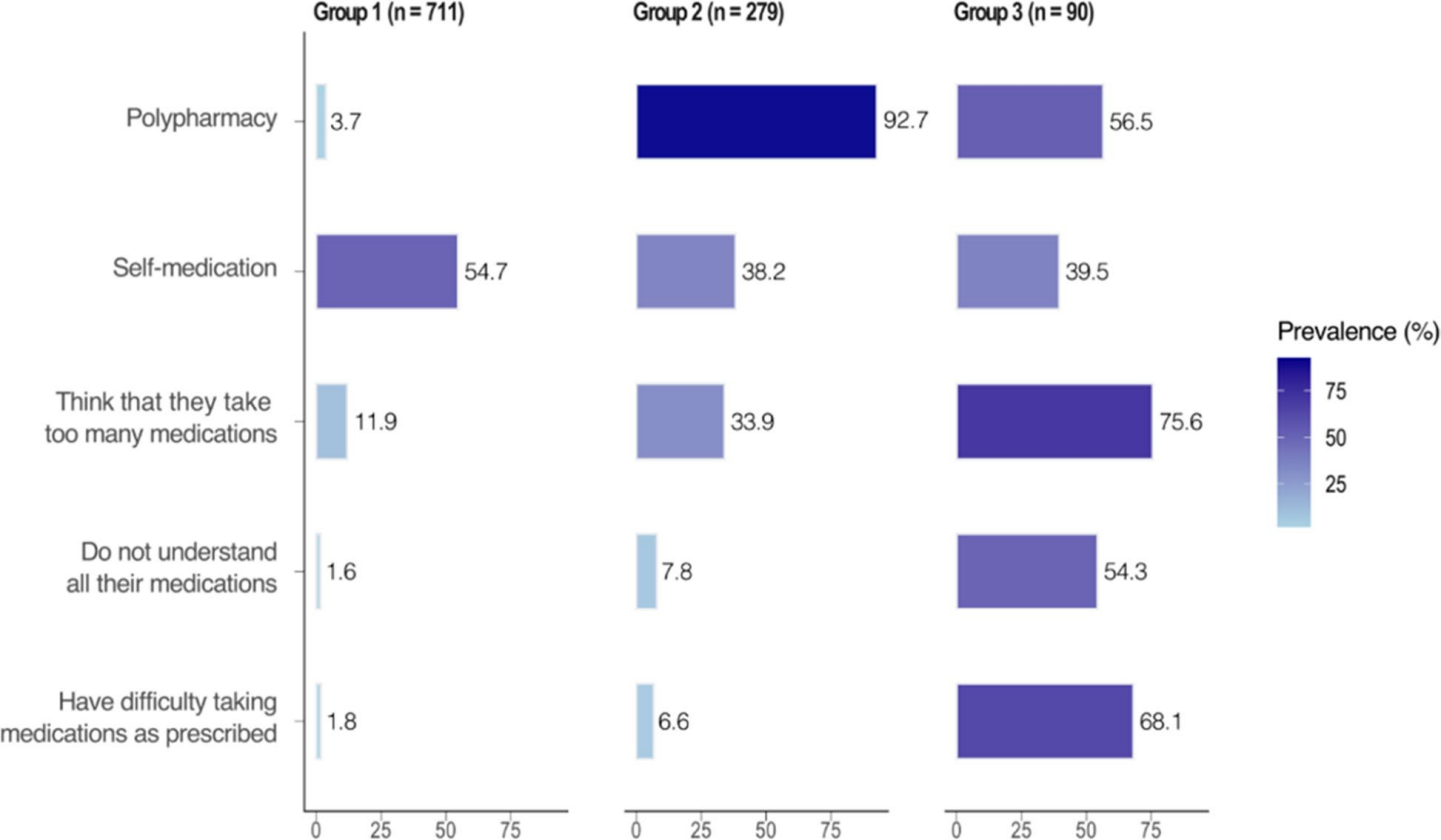
Profiles of the three patient groups derived from latent class analysis (model-based prevalence, %) (*N* = 1,080)

LCA yielded three groups among the respondents taking $\ge$ 2 medications accounting for 69.2% of the French older population. Figure 2 demonstrates three group profiles. We characterized them as follows: Group 1 (‘Non-polypharmacy, positive perceptions’, *n* = 711) – people who were more likely to take 2–4 medications concurrently and to have positive perceptions of their medications, Group 2 (‘Polypharmacy, positive perceptions’, *n* = 279) – people who were more likely to take $\ge$ 5 medications concurrently and to have positive perceptions of their medications, and Group 3 (‘Negative perceptions’, *n* = 90) – people who tended to think that they take too many medications (75.6%), that they do not understand all medications (54.3%), and that they have difficulty taking medications as prescribed (68.1%). There was no significant difference in the prevalence of self-medication among three groups, ranging from 38.2 to 54.7%. The three groups differed according to age, education, income, and all health-related factors (Supplementary File–Table S5). Table 3 showed that the assignment to Group 2 compared to Group 1 was associated with low education, presence of chronic conditions, activity limitation, and frailty. The assignment to Group 3 compared to Group 1 was associated with increasing age (80–85 years), low education, very bad/bad/fair self-perceived health, activity limitation, and frailty.

**Table 3:** Factors associated with group assignment (*n* = 954)

	**Model 1**		**Model 2**	
**Characteristics**	**Adjusted OR (95% CI)**		**Adjusted OR (95% CI)**	
	**Group 2/1**	**Group 3/1**	**Group 2/1**	**Group 3/1**
**Sociodemographic variables**				
*Sex*				
Male	Ref	Ref	Ref	Ref
Female	0.75 (0.47 – 1.18)	0.85 (0.44 – 1.62)	0.58 (0.35– 0.96) **	0.64 (0.32–1.27)
*Age group (years)*				
70 – 74	Ref	Ref	Ref	Ref
75 – 79	1.23 (0.79 – 1.93)	0.72 (0.34 – 1.49)	1.02 (0.62–1.70)	0.53 (0.24–1.18)
80 – 85	1.34 (0.84 – 2.15)	2.15 (1.16 – 3.98) **	1.12 (0.69–1.84)	1.69 (0.89–3.21)
*Living situation*				
Not living alone	Ref	Ref	Ref	Ref
Living alone	1.10 (0.71 – 1.69)	0.84 (0.46 – 1.55)	0.90 (0.55–1.48)	0.69 (0.37–1.29)
*Socio-professional category*				
Intermediate/Executive/Intellectual	Ref	Ref	Ref	Ref
Employees	0.78 (0.45 – 1.34)	1.28 (0.53 – 3.06)	0.70 (0.38–1.30)	1.10 (0.42–2.91)
Laborers/Farmers/Craftsmen/Traders	0.71 (0.43 – 1.19)	1.20 (0.58 – 2.49)	0.73 (0.43–1.23)	1.14 (0.53–2.44)
*Education level*				
None/Primary	Ref	Ref	Ref	Ref
Secondary	0.56 (0.36 – 0.86) ***	0.58 (0.32 – 1.07) *	0.56 (0.35–0.89) **	0.58 (0.31–1.11)
Post-secondary	0.35 (0.19 – 0.64) ***	1.16 (0.53 – 2.53)	0.33 (0.18–0.62) ***	0.99 (0.42–2.33)
*Income (euros)*				
< 1500	Ref	Ref	Ref	Ref
1500 – 3000	1.08 (0.67 – 1.75)	0.98 (0.50 – 1.92)	1.15 (0.67–1.99)	0.97 (0.49–1.91)
> 3000	0.85 (0.44 – 1.67)	0.29 (0.12 – 0.70) ***	0.91 (0.43–1.91)	0.28 (0.11–0.73) ***
**Health-related variables**				
*Self-perceived health*				
Very good/Good	-	-	Ref	Ref
Very bad/Bad/Fair			1.38 (0.89–2.14)	2.11 (1.00–4.44) **
*Chronic conditions*				
No	-	-	Ref	Ref
Yes			2.05 (1.28–3.26) ***	2.16 (0.90–5.16) *
*Activity limitation*				
Not limited	-	-	Ref	Ref
Limited but not severely			1.77 (1.07–2.91) **	2.83 (1.33–6.01) ***
Severely limited			2.55 (1.45–4.49) ***	2.62 (1.04–6.59) **
*Frailty status*				
Not frail	-	-	Ref	Ref
Pre-frail			0.95 (0.59–1.54)	1.57 (0.74–3.36)
Frail			3.02 (1.55–5.90) ***	3.37 (1.27–8.95) **

*OR* Odds Ratio

^*^ p < 0.1; ** p < 0.05; *** p < *0.01*

Model 1: adjusted for socio-demographic variables

Model 2: adjusted for socio-demographic and health-related variables

Our sensitivity analysis showed that the results obtained from the original and imputed datasets were similar with regards to the strength and the direction of associations (Supplementary File–Tables S6–S8).

## Discussion

We observed that 23.5% of older adults aged 70–85 years in France were on polypharmacy, while nearly 50% practiced self-medication, although occasionally in the vast majority of cases. Most older adults believed that they understood their medications and had no difficulty taking medications as prescribed. However, about 23% of those taking $\ge$ 2 medications thought they took too many medications. We also demonstrated a wide range of factors associated with polypharmacy, self-medication, and patient perceptions of medications. Furthermore, among older adults taking $\ge$ 2 medications, we distinguished three patient groups that differed in terms of polypharmacy and perceptions of medications.

First, polypharmacy was common among older adults in our study. However, the observed prevalence of polypharmacy (23.5%) is lower than those reported from previous studies conducted in France and in other developed countries [21–23]. This can be explained by the heterogeneous measurements of the number of medications taken and also by different study populations. Regarding the associated factors, our findings reinforce the associations of polypharmacy with age, education, and health-related factors [24–26]. More attention is needed to prevent inappropriate polypharmacy among older people with low education and impaired health status. We did not observe any association between sex and polypharmacy. Previous findings on the association between sex and polypharmacy are inconsistent. No association was found in the study of Slater et al., [27, 28] while other studies concluded positive associations of polypharmacy with female sex [29], or male sex [30].

Second, our results showed that self-medication was common in older adults. The prevalence of self-medication in our study (48.7%) is higher than the average prevalence in people aged $\ge$ 65 years from 14 European countries (26.3%) reported by Brandão et al. [31] The difference between the two results can be explained by varying data collection methods and patterns of self-medication across countries which depend on local healthcare policies and advertising regulations [32]. Consistent with prior studies, we found that women and adults with high education were more likely to self-medicate [11, 31]. Women have been described to be more interested in health-related information [33]; while people with high education tend to be well-informed by reliable sources, which probably make them feel more confident in purchasing nonprescription medications. The results suggest the necessity of conducting thorough interview and medication review, including nonprescription medications and dietary supplements used at home during medical consultations.

Third, regarding patient perceptions, women were seen to be less likely to think they take too many medications than men. One explanation could be that women tend to be more aware of their health conditions, visiting doctors more frequently than men to seek treatments [34, 35], thereby being more willing to take prescribed medications. The proportion of older adults reporting not understanding all medications increased with age and the number of medications taken. ‘Have difficulty taking medications as prescribed’ was associated with advancing age, low income, activity limitation, and frailty. This aligns with previous research showing that financial and physical constraints could prevent patients from taking medications as prescribed [36, 37]. However, since the French people have limited out-of-pocket expenses of medications [38], the underlying mechanism for the association with low income is uncertain. Although crude associations between three variables pertaining to socio-economic status (socio-professional category, education level, income) with the outcomes were found, only income remained significantly associated with ‘do not understand all medications’ and ‘have difficulty taking medications as prescribed’ in the multivariate analysis. Similarly, only education remained associated with polypharmacy and self-medication. This might reflect the complexity of evaluating social inequality in health risks at older ages [39]. Those associations thus need to be interpreted with caution.

Fourth, three patient groups were identified among older adults taking $\ge$ 2 medications, including Group 1 (‘Non-polypharmacy, positive perceptions’), Group 2 (‘Polypharmacy, positive perceptions’), and Group 3 (‘Negative perceptions’). Based on the results, we were able to distinguish people with negative perceptions of their medications from those with positive perceptions. Older adults in Group 3 were less likely to report polypharmacy but more likely to think that they take too many medications than those in Group 2. This underlines the difference between the objective measure of polypharmacy ($\ge$ 5 medications) and the perceived measure defined by the variable ‘think they take too many medications’. In health research, the subjective assessments have been shown to aid in capturing multiple-faceted concerns along with the objective assessments and predicting health outcomes [40]. It is thus essential to take the subjective measure of polypharmacy into account by questioning about patient perceptions in future research. Regarding the factors associated with the assignment to Group 2 and Group 3 compared to Group 1, some factors were found to be significantly associated but no major differences emerged. Similar to patient perceptions of health status [41], perceptions of medications might be influenced by a complex set of factors. These factors could include not only some patient-related factors which we observed but also treatment-related factors, pathologies, and physician-related factors which we did not assess in this study.

The strength of our study is that we used data from a nationally representative survey – the ‘French Health Barometer’. Although we did not use validated tools due to the limited duration of the survey interview, the survey questions used to explore patient perceptions of medications are similar to questions in validated questionnaires [42]. Our study has some limitations. First, we studied people in a limited age range (70–85 years) which prevents us from studying the phenomena at older age (> 85 years). Second, reported information on the number of medications taken and self-medication could be threatened by recall bias. Third, we did not have information on the assistance on preparing or taking medications, which can confound the associations of our outcomes with some factors. Finally, there was a lack of information on the therapeutic regimen complexity, the pathologies, and the physician–patient relationship that could have provided clues for a more accurate interpretation of the studied phenomena. These factors should be examined in future studies to develop a deeper understanding of patient perceptions.

## Conclusion

Polypharmacy and self-medication are common in the French population aged 70–85 years. Most of older adults reported positive perceptions of their medications. However, there remained a group of older adults who tended to think that they take too many medications regardless of their polypharmacy status, having difficulty understanding and taking their medications as prescribed. The findings suggest the importance of the perceived measure of polypharmacy in research, which makes possible to identify difficulties and support needs in people who are not considered to be at risk according to objective polypharmacy indicators.

## Supplementary Information

**Additional file 1: Table S1. **List of the variables composing the frailty phenotype. **Table S2.** Factors associated with ‘Think that they take too many medications’. Model 1: adjusted for socio-demographic variables. Model 2: adjusted for socio-demagraphic and health-related variables. **Table S3.** Factors associated with ‘Think they do not understand all their medications’. Model 1: adjusted for socio-demographic variables. Model 2: adjusted for socio-demagraphic and health-related variables. **Table S4.** Factors associated with ‘Have difficulty taking their medications as prescribed’. Model 1: adjusted for socio-demographic variables. Model 2: adjusted for socio-demagraphic and health-related variables. **Table S5.** Statistical description of three patient groups, n (weighted percentages) (*n*=1,080). **Table S6.** Factors associated with polypharmacy and self–medication (results from imputed dataset). Model 1: adjusted for socio-demographic variables. Model 2: adjusted for socio-demagraphic and health-related variables. **Table S7.** Factors associated with three outcomes pertaining to patient perceptions of medications (results from imputed dataset). Model 1: adjusted for socio-demographic variables. Model 2: adjusted for socio-demagraphic and health-related variables. **Table S8.** Factors associated with group assignment (*n*=1,080) (results from imputed dataset). Model 1: adjusted for socio-demographic variables. Model 2: adjusted for socio-demagraphic and health-related variables.

## Data Availability

The datasets generated during and analyzed during the current study are not publicly available but are available from the corresponding author (laure.carcaillon-bentata@santepubliquefrance.fr) on reasonable request.
