# Supplementary material for: Uses and perceptions of medications among French older adults: results from the 2020 French Health Barometer survey
Source: BMC Geriatr. 2022 Jul 20;22:602. doi: 10.1186/s12877-022-03289-9 (PMC9301842; doi:10.1186/s12877-022-03289-9)
Supplement: Supplementary file 1 — Additional file 1: Table S1. List of the variables composing the frailty phenotype. Table S2. Factors associated with ‘Think that they take too many medications’. Model 1: adjusted for socio-demographic variables. Model 2: adjusted for socio-demagraphic and health-related variables. Table S3. Factors associated with ‘Think they do not understand all their medications’. Model 1: adjusted for socio-demographic variables. Model 2: adjusted for socio-demagraphic and health-related variables. Table S4. Factors associated with ‘Have difficulty taking their medications as prescribed’. Model 1: adjusted for socio-demographic variables. Model 2: adjusted for socio-demagraphic and health-related variables. Table S5. Statistical description of three patient groups, n (weighted percentages) (n=1,080). Table S6. Factors associated with polypharmacy and self–medication (results from imputed dataset). Model 1: adjusted for socio-demographic variables. Model 2: adjusted for socio-demagraphic and health-related variables. Table S7. Factors associated with three outcomes pertaining to patient perceptions of medications (results from imputed dataset). Model 1: adjusted for socio-demographic variables. Model 2: adjusted for socio-demagraphic and health-related variables. Table S8. Factors associated with group assignment (n=1,080) (results from imputed dataset). Model 1: adjusted for socio-demographic variables. Model 2: adjusted for socio-demagraphic and health-related variables. [file 12877_2022_3289_MOESM1_ESM.pdf]

Supplementary File

Table S1. List of the variables composing the frailty index

Component	Question(s)	Component coding
**Exhaustion**	Q1. Over the last month, have you lacked the energy to do the things you wanted to do?<br>1. Yes<br>2. No	1: if the individual reports having lacked energy<br>0: otherwise
**Unintentional weight loss**	Q1. Have you involuntarily lost weight over the last 12 months, outside any form of diet?<br>1. Yes<br>2. No<br>*If (Q1=1):*<br>Q2. How much weight have you lost in kilos?	1: if weight loss is greater than a 5% loss of original weight.<br>0: otherwise
**Muscle weakness**	Q1. Do you have difficulty carrying a 5kg bag, such as a heavy bag of groceries, without help?<br>Q2. Do you have difficulty using your hands and fingers without technical assistance?<br>Q3. Do you have difficulty bending or kneeling down without help?<br>*For each question:*<br>1. No difficulty<br>2. Some difficulty<br>3. A great deal of difficulty<br>4. Incapable	1: if the individual reports having a great deal of difficulty (3) or if he/she considers himself/herself incapable to carrying out (4) at least one of the two activities mentioned.<br>0: otherwise.<br>*If the individual reports having difficulty carrying a 5kg bag, he/she must report not having difficulties using hands and fingers for the response to be valid.*
**Impaired mobility**	Q1. Do you have difficulty walking 500 meters without help?<br>Q2. Do you have difficulty going up or down a dozen or more steps without help?<br>*For each question:*<br>1. No difficulty<br>2. Some difficulty<br>3. Great difficulty<br>4. Incapable	1: if the individual reports a great deal of difficulty (3) or if he/she considers being incapable (4) of carrying out at least one of the two activities mentioned.<br>0: otherwise
**Low physical activity**	Q1. How often do you take part in activities requiring moderate physical efforts, such as gardening, cleaning the car or going for a walk? 1. Several times a week<br>2. Once a week<br>3. One to three times a week<br>4. Rarely or never	1: if the individual reports more than once a week<br>0: otherwise

Supplementary File

Table S2. Factors associated with 'Think that they take too many medications'

Characteristics	Think they take too many medications		
	Crude PR (95% CI)	Model 1 (n=950) Adjusted PR (95% CI)	Model 2 (n=950) Adjusted PR (95% CI)
**Sociodemographic variables**			
Sex			
Male	Ref	Ref	Ref
Female	0.72 (0.55 – 0.94) **	0.66 (0.48 – 0.92) **	0.66 (0.47 – 0.92) **
Age group (years)			
70 – 74	Ref	Ref	Ref
75 – 79	0.76 (0.55 – 1.04) *	0.80 (0.57 – 1.12)	0.80 (0.57 – 1.13)
80 – 85	0.80 (0.56 – 1.13)	0.70 (0.48 – 1.02) *	0.69 (0.48 – 0.98) **
Living situation			
Not living alone	Ref	Ref	Ref
Living alone	0.96 (0.73 – 1.25)	0.91 (0.66 – 1.25)	0.89 (0.66 – 1.21)
Socio – professional category			
Intermediate/Executive/Intellectual	Ref	Ref	Ref
Employees	0.87 (0.61 – 1.23)	0.93 (0.60 – 1.42)	1.00 (0.66 – 1.52)
Laborers/Farmers/Craftsmen/Traders	1.08 (0.80 – 1.47)	0.88 (0.61 – 1.27)	0.90 (0.64 – 1.29)
Education level			
None/Primary	Ref	Ref	Ref
Secondary	0.89 (0.66 – 1.21)	0.84 (0.61 – 1.15)	0.97 (0.70 – 1.35)
Post – secondary	0.83 (0.59 – 1.18)	0.77 (0.50 – 1.18)	0.88 (0.58 – 1.35)
Income (euros)			
< 1500	Ref	Ref	Ref
1500 – 3000	0.96 (0.69 – 1.32)	0.90 (0.63 – 1.28)	0.96 (0.68 – 1.37)
> 3000	0.87 (0.59 – 1.29)	0.75 (0.46 – 1.23)	0.82 (0.50 – 1.35)
**Health – related variables**			
Self – perceived health			
Very good/Good	Ref	–	Ref
Very bad/Bad/Fair	1.39 (1.05 – 1.84) **		0.87 (0.64 – 1.17)
Chronic conditions			
No	Ref	–	Ref
Yes	1.65 (1.15 – 2.38) ***		1.32 (0.92 – 1.90)
Activity limitation			
Not limited	Ref	–	Ref
Limited but not severely	1.69 (1.24 – 2.31) ***		1.31 (0.92 – 1.87)
Severely limited	1.60 (1.12 – 2.27) ***		0.99 (0.63 – 1.58)
Frailty status			
Not frail	Ref	–	Ref
Pre – frail	1.33 (0.95 – 1.85) *		1.19 (0.85 – 1.66)
Frail	1.83 (1.25 – 2.70) ***		1.10 (0.65 – 1.88)
**Number of medications taken**	1.11 (1.07 – 1.15) ***	–	1.16 (1.11 – 1.20) ***

PR = Prevalence Ratio

** p < 0.1; ** p < 0.05; *** p < 0.01*

Supplementary File

Table S3. Factors associated with 'Think they do not understand all their medications'

Characteristics	Think they do not understand all their medications		
		**Model 1 (n=1,170)**	**Model 2 (n=1,170)**
	**Crude PR (95% CI)**	**Adjusted PR (95% CI)**	**Adjusted PR (95% CI)**
**Sociodemographic variables**			
Sex			
Male	Ref	Ref	Ref
Female	1.18 (0.73 – 1.93)	0.82 (0.44 – 1.52)	0.80 (0.45 – 1.43)
Age group (years)			
70 – 74	Ref	Ref	Ref
75 – 79	1.62 (0.88 – 2.98)	1.29 (0.68 – 2.45)	1.24 (0.62 – 2.49)
80 – 85	2.50 (1.45 – 4.29) ***	1.96 (1.12 – 3.42) **	1.82 (1.04 – 3.17) **
Living situation			
Not living alone	Ref	Ref	Ref
Living alone	1.36 (0.83 – 2.23)	1.14 (0.59 – 2.18)	1.11 (0.60 – 2.05)
Socio – professional category			
Intermediate/Executive/Intellectual	Ref	Ref	Ref
Employees	1.37 (0.71 – 2.65)	0.99 (0.43 – 2.25)	0.98 (0.44 – 2.18)
Laborers/Farmers/Craftsmen/Traders	2.32 (1.36 – 3.97) ***	1.48 (0.82 – 2.67)	1.38 (0.79 – 2.41)
Education level			
None/Primary	Ref	Ref	Ref
Secondary	0.51 (0.31 – 0.86) **	0.76 (0.45 – 1.28)	0.84 (0.49 – 1.43)
Post – secondary	0.47 (0.25 – 0.86) **	0.96 (0.48 – 1.93)	1.01 (0.50 – 2.06)
Income (euros)			
< 1500	Ref	Ref	Ref
1500 – 3000	0.61 (0.35 – 1.06) *	0.73 (0.38 – 1.37)	0.80 (0.43 – 1.51)
> 3000	0.31 (0.15 – 0.62) ***	0.38 (0.17 – 0.86) **	0.42 (0.18 – 0.97) **
**Health – related variables**			
Self – perceived health			
Very good/Good	Ref	–	Ref
Very bad/Bad/Fair	2.03 (1.19 – 3.47) **		1.01 (0.55 – 1.84)
Chronic conditions			
No	Ref	–	Ref
Yes	1.59 (0.87 – 2.92)		1.33 (0.64 – 2.75)
Activity limitation			
Not limited	Ref	–	Ref
Limited but not severely	2.93 (1.67 – 5.15) ***		1.82 (0.90 – 3.65) *
Severely limited	2.54 (1.32 – 4.91) ***		1.10 (0.44 – 2.80)
Frailty status			
Not frail	Ref	–	Ref
Pre – frail	1.65 (0.84 – 3.23)		1.05 (0.53 – 2.08)
Frail	3.50 (1.75 – 7.01) ***		1.04 (0.41 – 2.62)
**Number of medications taken**	1.14 (1.11 – 1.18) ***	–	1.16 (1.08 – 1.25) ***

PR = Prevalence Ratio

** p < 0.1; ** p < 0.05; *** p < 0.01*

Supplementary File

Table S4. Factors associated with 'Have difficulty taking their medications as prescribed'

Characteristics	Have difficulty taking their medications as prescribed		
	Crude PR (95% CI)	Model 1 (n=1,170) Adjusted PR (95% CI)	Model 2 (n=1,170) Adjusted PR (95% CI)
**Sociodemographic variables**			
Sex			
Male	Ref	Ref	Ref
Female	1.58 (1.01 – 2.47) **	0.95 (0.58 – 1.57)	0.82 (0.51 – 1.32)
Age group (years)			
70 – 74	Ref	Ref	Ref
75 – 79	0.99 (0.56 – 1.76)	0.77 (0.41 – 1.42)	0.62 (0.34 – 1.14)
80 – 85	2.07 (1.27 – 3.39) ***	1.67 (1.02 – 2.72) **	1.33 (0.84 – 2.11)
Living situation			
Not living alone	Ref	Ref	Ref
Living alone	0.89 (0.56 – 1.41)	0.82 (0.50 – 1.33)	0.72 (0.45 – 1.14)
Socio – professional category			
Intermediate/Executive/Intellectual	Ref	Ref	Ref
Employees	2.44 (1.40 – 4.26) ***	1.87 (0.97 – 3.62) *	1.77 (0.92 – 3.43) *
Laborers/Farmers/Craftsmen/Traders	1.78 (1.04 – 3.05) **	1.48 (0.82 – 2.67)	1.37 (0.79 – 2.40)
Education level			
None/Primary	Ref	Ref	Ref
Secondary	0.42 (0.26 – 0.70) ***	0.62 (0.37 – 1.04) *	0.70 (0.42 – 1.17)
Post – secondary	0.46 (0.27 – 0.78) ***	1.32 (0.70 – 2.46)	1.34 (0.73 – 2.48)
Income (euros)			
< 1500	Ref	Ref	Ref
1500 – 3000	1.04 (0.63 – 1.71)	1.25 (0.75 – 2.08)	1.22 (0.76 – 1.97)
> 3000	0.23 (0.11 – 0.49) ***	0.31 (0.14 – 0.69) ***	0.32 (0.14 – 0.70) ***
**Health – related variables**			
Self – perceived health			
Very good/Good	Ref	–	Ref
Very bad/Bad/Fair	2.92 (1.76 – 4.84) ***		1.35 (0.78 – 2.34)
Chronic conditions			
No	Ref	–	Ref
Yes	2.64 (1.36 – 5.11) ***		1.93 (0.95 – 3.92) *
Activity limitation			
Not limited	Ref	–	Ref
Limited but not severely	3.77 (2.18 – 6.53) ***		2.57 (1.44 – 4.59) ***
Severely limited	4.63 (2.54 – 8.45) ***		2.07 (0.91 – 4.73) *
Frailty status			
Not frail	Ref	–	Ref
Pre – frail	2.47 (1.29 – 4.74) ***		2.00 (1.10 – 3.63) **
Frail	5.67 (2.90 – 11.08) ***		2.15 (0.99 – 4.66) *
**Number of medications taken**	1.12 (1.09 – 1.15) ***	–	1.04 (0.98 – 1.11)

PR = Prevalence Ratio

** p < 0.1; ** p < 0.05; *** p < 0.01*

Supplementary File

Table S5. Statistical description of three patient groups, n (weighted percentages) (n=1,080)

**Characteristics**	**Group 1 (62.5%)**	**Group 2 (28.0%)**	**Group 3 (9.5%)**	**p – value**
**Sociodemographic variables**				
Sex				0.250
Male	305 (46.0)	134 (49.7)	43 (37.8)	
Female	406 (54.0)	145 (50.3)	47 (62.2)	
Age group (years)				0.001
70 – 74	332 (43.7)	104 (35.4)	36 (29.8)	
75 – 79	222 (32.2)	90 (36.0)	22 (22.1)	
80 – 85	157 (24.1)	85 (28.6)	32 (48.1)	
Living situation				0.471
Not living alone	397 (65.2)	135 (60.1)	47 (62.6)	
Living alone	314 (34.8)	144 (39.9)	43 (37.4)	
Socio – professional category				0.617
Intermediate/Executive/Intellectual	326 (36.3)	130 (34.6)	34 (25.9)	
Employees	177 (27.8)	66 (30.2)	19 (33.4)	
Laborers/Farmers/Craftsmen/Traders	196 (35.9)	75 (35.2)	32 (40.8)	
Education level				< 0.001
None/Primary	175 (35.0)	107 (51.1)	35 (60.8)	
Secondary	316 (46.2)	113 (37.8)	33 (25.6)	
Post – secondary	214 (18.8)	55 (11.1)	21 (13.6)	
Income (euros)				0.018
< 1500	175 (31.6)	88 (36.7)	28 (42.4)	
1500 – 3000	296 (42.9)	119 (45.1)	47 (49.5)	
> 3000	173 (25.5)	52 (18.2)	11 (8.1)	
**Health – related variables**				
Self – reported health				< 0.001
Very good/Good	409 (57.7)	91 (33.1)	21 (23.0)	
Very bad/Bad/Fair	299 (42.3)	188 (66.9)	69 (77.0)	
Chronic disease				< 0.001
No	258 (39.0)	48 (19.2)	15 (19.9)	
Yes	448 (61.0)	230 (80.8)	74 (80.1)	
Activity limitations				< 0.001
Not limited	432 (62.3)	97 (35.6)	24 (25.0)	
Limited but not severely	192 (26.3)	83 (31.0)	41 (46.7)	
Severely limited	84 (11.4)	97 (33.4)	25 (28.3)	
Frailty status				< 0.001
Not frail	309 (42.5)	71 (26.2)	17 (17.4)	
Pre – frail	344 (48.5)	130 (41.3)	44 (45.9)	
Frail	56 (9.0)	78 (32.5)	29 (36.7)	

Supplementary File

Table S6. Factors associated with polypharmacy and self–medication (results from imputed dataset)

Characteristics	Polypharmacy		Self – medication	
	Model 1 (n=1,614) PR adjusted (95% CI)	Model 2 (n=1,614) PR adjusted (95% CI)	Model 1 (n=1,620) PR adjusted (95% CI)	Model 2 (n=1,620) PR adjusted (95% CI)
**Sociodemographic variables**				
Sex				
Male	Ref	Ref	Ref	Ref
Female	0.74 (0.56 – 0.96) **	0.61 (0.47 – 0.79) ***	1.21 (1.06 – 1.39) ***	1.22 (1.07 – 1.40) ***
Age group (years)				
70 – 74	Ref	Ref	Ref	Ref
75 – 79	1.34 (1.02 – 1.76) **	1.11 (0.85 – 1.45)	1.00 (0.88 – 1.15)	1.03 (0.90 – 1.17)
80 – 85	1.32 (0.99 – 1.77) *	1.10 (0.86 – 1.42)	0.86 (0.72 – 1.02) *	0.89 (0.75 – 1.05)
Living situation				
Not living alone	Ref	Ref	Ref	Ref
Living alone	1.18 (0.92 – 1.51)	1.02 (0.81 – 1.28)	1.03 (0.90 – 1.20)	1.08 (0.93 – 1.24)
Socio – professional category				
Intermediate/Executive/Intellectual	Ref	Ref	Ref	Ref
Employees	0.97 (0.70 – 1.35)	0.95 (0.70 – 1.30)	1.10 (0.93 – 1.30)	1.11 (0.94 – 1.30)
Laborers/Farmers/Craftsmen/Traders	0.93 (0.68 – 1.26)	0.94 (0.72 – 1.22)	0.98 (0.82 – 1.17)	0.97 (0.82 – 1.16)
Education level				
None/Primary	Ref	Ref	Ref	Ref
Secondary	0.65 (0.51 – 0.84) ***	0.69 (0.55 – 0.88) ***	1.32 (1.11 – 1.56) ***	1.30 (1.10 – 1.53) ***
Post – secondary	0.51 (0.35 – 0.74) ***	0.54 (0.38 – 0.76) ***	1.54 (1.25 – 1.88) ***	1.49 (1.23 – 1.82) ***
Income (euros)				
< 1500	Ref	Ref	Ref	Ref
1500 – 3000	0.83 (0.62 – 1.10)	0.86 (0.66 – 1.12)	1.03 (0.85 – 1.25)	1.01 (0.84 – 1.21)
> 3000	0.71 (0.47 – 1.07)	0.76 (0.50 – 1.16)	1.25 (0.98 – 1.59) *	1.24 (0.98 – 1.56) *
**Health – related variables**				
Self – reported health				
Very good/Good	–	Ref	–	Ref
Very bad/Bad/Fair		1.56 (1.17 – 2.08) ***		1.03 (0.90 – 1.18)
Chronic disease				
No	–	Ref	–	Ref
Yes		2.41 (1.77 – 3.30) ***		0.93 (0.81 – 1.05)
Activity limitations				
Not limited	–	Ref	–	Ref
Limited but not severely		1.56 (1.14 – 2.12) ***		1.28 (1.12 – 1.47) ***
Severely limited		1.91 (1.39 – 2.62) ***		0.87 (0.68 – 1.11)
Frailty status				
Not frail	–	Ref	–	Ref
Pre – frail		1.06 (0.78 – 1.43)		1.01 (0.89 – 1.14)
Frail		1.74 (1.23 – 2.46) ***		0.70 (0.52 – 0.93) **

PR = Prevalence Ratio

** p < 0.1; ** p < 0.05; *** p < 0.01*

Supplementary File

Table S7. Factors associated with three outcomes pertaining to patient perceptions of medications (results from imputed dataset)

Characteristics	Think they take too many medications		Think they do not understand all their medications		Have difficulty taking their medications as prescribed	
	**Model 1 (n=1,074) PR adjusted (95% CI)**	**Model 2 (n=1,074) PR adjusted (95% CI)**	**Model 1 (n=1,317) PR adjusted (95% CI)**	**Model 2 (n=1,317) PR adjusted (95% CI)**	**Model 1 (n=1,319) PR adjusted (95% CI)**	**Model 2 (n=1,319) PR adjusted (95% CI)**
**Sociodemographic variables**						
Sex						
Male	Ref	Ref	Ref	Ref	Ref	Ref
Female	0.68 (0.50 – 0.93) **	0.62 (0.46 – 0.85) ***	1.07 (0.62 – 1.86)	0.96 (0.56 – 1.66)	1.17 (0.72 – 1.89)	0.97 (0.59 – 1.61)
Age group (years)						
70 – 74	Ref	Ref	Ref	Ref	Ref	Ref
75 – 79	0.76 (0.55 – 1.05) *	0.74 (0.54 – 1.02) *	1.38 (0.76 – 2.50)	1.33 (0.71 – 2.48)	0.89 (0.51 – 1.54)	0.74 (0.43 – 1.27)
80 – 85	0.76 (0.54 – 1.07)	0.65 (0.47 – 0.90) **	2.15 (1.28 – 3.61) ***	1.77 (1.05 – 2.98) **	1.77 (1.10 – 2.83) **	1.42 (0.88 – 2.31)
Living situation						
Not living alone	Ref	Ref	Ref	Ref	Ref	Ref
Living alone	0.97 (0.72 – 1.32)	1.02 (0.77 – 1.35)	0.96 (0.54 – 1.71)	1.07 (0.63 – 1.90)	0.65 (0.41 – 1.03) *	0.62 (0.40 – 0.96) **
Socio – professional category						
Intermediate/Executive/Intellectual	Ref	Ref	Ref	Ref	Ref	Ref
Employees	0.92 (0.61 – 1.39)	0.98 (0.64 – 1.49)	0.92 (0.43 – 1.96)	0.87 (0.41 – 1.85)	1.94 (0.99 – 3.79) *	1.93 (0.97 – 3.82) *
Laborers/Farmers/Craftsmen/Traders	0.94 (0.66 – 1.34)	0.96 (0.68 – 1.34)	1.66 (0.91 – 3.01) *	1.52 (0.86 – 2.66)	1.51 (0.81 – 2.83)	1.44 (0.77 – 2.68)
Education level						
None/Primary	Ref	Ref	Ref	Ref	Ref	Ref
Secondary	0.84 (0.62 – 1.12)	0.96 (0.71 – 1.30)	0.74 (0.45 – 1.20)	0.83 (0.50 – 1.37)	0.55 (0.33 – 0.91) **	0.62 (0.38 – 1.01) *
Post – secondary	0.79 (0.52 – 1.19)	0.87 (0.58 – 1.31)	0.94 (0.49 – 1.82)	0.98 (0.50 – 1.92)	1.14 (0.60 – 2.18)	1.18 (0.62 – 2.23)
Income (euros)						
< 1500	Ref	Ref	Ref	Ref	Ref	Ref
1500 – 3000	0.88 (0.62 – 1.25)	0.99 (0.70 – 1.40)	0.68 (0.36 – 1.28)	0.82 (0.43 – 1.54)	1.21 (0.71 – 2.04)	1.33 (0.81 – 2.19)
> 3000	0.74 (0.45 – 1.19)	0.85 (0.52 – 1.39)	0.40 (0.18 – 0.88) **	0.51 (0.22 – 0.17) **	0.31 (0.12 – 0.77) **	0.36 (0.15 – 0.89) **
**Health – related variables**						
Self – reported health						
Very good/Good	–	Ref	–	Ref	–	Ref
Very bad/Bad/Fair		0.99 (0.74 – 1.33)		1.06 (0.62 – 1.83)		1.36 (0.83 – 2.25)
Chronic disease						
No	–	Ref	–	Ref	–	Ref
Yes		1.34 (0.95 – 1.88) *		1.06 (0.56 – 2.03)		1.78 (0.93 – 3.41) *
Activity limitations						
Not limited	–	Ref	–	Ref	–	Ref
Limited but not severely		1.37 (0.98 – 1.91) *		2.06 (1.11 – 3.80) **		2.17 (1.30 – 3.64) ***
Severely limited		0.98 (0.64 – 1.50)		0.93 (0.37 – 2.33)		1.99 (0.93 – 4.23) *
Frailty status						
Not frail	–	Ref	–	Ref	–	Ref
Pre – frail		1.27 (0.92 – 1.74)		1.23 (0.64 – 2.38)		1.47 (0.80 – 2.73)
Frail		1.32 (0.82 – 2.14)		1.54 (0.65 – 3.62)		1.78 (0.85 – 3.73) *
**Number of medications taken**	–	1.12 (1.08 – 1.16) ***		1.11 (1.05 – 1.18) ***		1.04 (1.00 – 1.08) **

PR = Prevalence Ratio

** p < 0.1; ** p < 0.05; *** p < 0.01*

Supplementary File

Table S8. Factors associated with group assignment (n=1,080) (results from imputed dataset)

**Characteristics**	**Model 1 OR adjusted (95% CI)**		**Model 2 OR adjusted (95% CI)**	
	**Group 2/1**	**Group 3/1**	**Group 2/1**	**Group 3/1**
**Sociodemographic variables**				
Sex				
Male	Ref	Ref	Ref	Ref
Female	0.64 (0.42 – 0.99) **	1.06 (0.57 – 1.98)	0.49 (0.31 – 0.79) ***	0.80 (0.39 – 1.65)
Age group (years)				
70 – 74	Ref	Ref	Ref	Ref
75 – 79	1.36 (0.90 – 2.07)	0.87 (0.45 – 1.69)	1.16 (0.73 – 1.84)	0.69 (0.34 – 1.42)
80 – 85	1.22 (0.78 – 1.92)	2.46 (1.37 – 4.42) ***	1.05 (0.65 – 1.69)	1.98 (1.03 – 3.78) **
Living situation				
Not living alone	Ref	Ref	Ref	Ref
Living alone	1.23 (0.82 – 1.83)	0.73 (0.40 – 1.31)	1.08 (0.69 – 1.68)	0.67 (0.37 – 1.21)
Socio – professional category				
Intermediate/Executive/Intellectual	Ref	Ref	Ref	Ref
Employees	0.89 (0.53 – 1.48)	1.27 (0.53 – 3.05)	0.87 (0.49 – 1.55)	1.15 (0.42 – 3.16)
Laborers/Farmers/Craftsmen/Traders	0.68 (0.42 – 1.11)	1.19 (0.54 – 2.61)	0.72 (0.44 – 1.19)	1.14 (0.49 – 2.66)
Education level				
None/Primary	Ref	Ref	Ref	Ref
Secondary	0.52 (0.34 – 0.79) ***	0.43 (0.24 – 0.75) ***	0.52 (0.33 – 0.81) ***	0.44 (0.24 – 0.80) ***
Post – secondary	0.35 (0.20 – 0.62) ***	0.97 (0.46 – 2.09)	0.33 (0.18 – 0.60) ***	0.82 (0.35 – 1.91)
Income (euros)				
< 1500	Ref	Ref	Ref	Ref
1500 – 3000	1.05 (0.66 – 1.65)	0.92 (0.46 – 1.85)	1.14 (0.69 – 1.89)	0.98 (0.47 – 2.02)
> 3000	0.84 (0.44 – 1.60)	0.26 (0.11 – 0.63) ***	0.90 (0.44 – 1.85)	0.29 (0.11 – 0.73) ***
**Health – related variables**				
Self – reported health				
Very good/Good	–	–	Ref	Ref
Very bad/Bad/Fair			1.42 (0.93 – 2.15)	2.14 (1.10 – 4.16) **
Chronic disease				
No	–	–	Ref	Ref
Yes			2.29 (1.47 – 3.58) ***	2.08 (0.93 – 4.68) *
Activity limitations				
Not limited	–	–	Ref	Ref
Limited but not severely			1.49 (0.92 – 2.41)	2.36 (1.20 – 4.63) **
Severely limited			2.57 (1.49 – 4.40) ***	2.17 (0.91 – 5.19) *
Frailty status				
Not frail	–	–	Ref	Ref
Pre – frail			0.91 (0.58 – 1.43)	1.23 (0.58 – 2.64)
Frail			2.48 (1.30 – 4.70) ***	2.84 (1.12 – 7.20) **

OR = Odds Ratio

** p < 0.1; ** p < 0.05; *** p < 0.01*
